# Inhaler Use Technique in Chronic Obstructive Pulmonary Disease Patients: Errors, Practices and Barriers

**DOI:** 10.7759/cureus.10569

**Published:** 2020-09-21

**Authors:** Tareen Sanaullah, Shereen Khan, Aria Masoom, Zahir K Mandokhail, Aisha Sadiqa, Muhammad Irfan Malik

**Affiliations:** 1 Pulmonary Medicine and Critical Care, FJ Chest Hospital, Bolan University of Medical and Health Sciences, Quetta, PAK; 2 Pulmonary Medicine and Critical Care, Bolan University of Medical and Health Sciences, Quetta, PAK; 3 Otolaryngology - Head and Neck Surgery, Bolan Medical Complex Hospital, Quetta, PAK; 4 Internal Medicine, Bolan Medical Complex Hospital, Quetta, PAK; 5 Gynaecology & Obstetrics, Civil Provincial Hospital, Quetta, PAK; 6 Pulmonology, Postgraduate Medical Institute/Ameer-ud-Din Medical College (AMC) Lahore General Hospital, Lahore, PAK

**Keywords:** inhaler technique, inhalation errors, barriers, mdi, dpi, copd, practices

## Abstract

Background

Inhaled medications are the main therapeutic treatment of chronic obstructive pulmonary disease (COPD) and inhaler technique remained important that can increase medication efficacy, reducing dose and side effects. Poor inhaler technique is multi-factorial and the quality of inhaler technique has not previously assessed in Pakistan. We conducted a study to examine a range of competing factors that impact COPD patient willingness, practices, and preference in using their inhalers.

Methods

A cross-sectional of 765 patients with COPD were interviewed and assessed by qualitative questionnaires. Objective inhalation technique and steps assessment was performed; satisfaction, preferences, perception, and practice of different types of inhaler devices were evaluated at a single cross-sectional visit at the study enrolment.

Results

The study included 765 participants of mean age 58.7 years (SD ±7.8); 32% males and 68% females. Almost all of the females were exposed to biomass fuel smoke exposure (99%) and pipe (Huka) smokers 53%, while most male participants were cigarette smokers (92%). Only 6.3% of participants were able to perform correct steps of inhaler use, and few educated patients completed 7-steps. 66% of patients were using dry powder inhalers (DPI) inhaler devices and mostly performed the steps 1, 2, and 4 (98%) correctly, while 44% who were using metered-dose inhalers (MDI) completed only steps 2 and 4 correctly (88%). The majority of participants reported the particular inhaler devices was prescribed by the visiting consultants (54%). Interestingly, they were using two inhalers together (47%) relieving symptoms of dyspnea (83%) and cough (73%). The inhaler use technique was demonstrated to most of the patients by the pharmacy salesman (38.4%), while 15.8% reported that their doctors taught them the inhaler technique. 54.2% reported reason for poor adherence to inhaler use as they understand it might not work lately and 75.2% were not aware of any side effects associated with the regular use of an inhaler.

Conclusions

Poor inhaler technique is highly prevalent and the associated errors did not appear to be dependent on device type. Most of the participants had not receive proper training about the correct use and were not involved in decision making about the choice of inhaler device.

## Introduction

Inhaled medications are the main therapeutic treatment of chronic obstructive pulmonary disease (COPD) with inhaler devices being the principal route for administering such treatments [1-2]. These treatments are given in the form of therapies that are emitted via nebulizers, pressurized metered-dose inhalers (pMDI), dry powder inhalers (DPI), or Soft Mist inhalers [3]. Each device has different operating, maintenance instructions, and successful use. A given inhaler requires that patients understand and maintain its proper use to ensure consistent optimal drug delivery into lower respiratory airways required by COPD patients.

Poor inhaler technique remains an important and multi-factorial problem that can stem from the device itself, the patient, the healthcare provider, technology, and policy [4]. Many studies concluded that a large numbers of COPD patients do not use their inhaler devices correctly. Errors in device use may impact the effectiveness of the drug delivery, thereby leading to the sub-optimal treatment of COPD that causes multiple episodes of acute exacerbation which is associated with numbers of morbidities and mortalities [5]. After recognizing these particular factors and challenges, we can successfully train most patients to optimaly use inhalers with effective and repeated instruction [6].

Since COPD is a chronic condition, the therapeutic adherence of inhaler and optimal use in routine with the correct technique is the cornerstone in the management. It is therefore imperative to understand and quantify device-use errors so that patient interventions can be effectively introduced and new devices designed to avoid common errors associated with routine use of inhalers and to complete delivery of inhaled medication. It is important to recognize these particular factors and errors which are complex, but yet evidence suggests that these challenges can be addressed [7].

## Materials and methods

This cross-sectional study was conducted from January 2018 to December 2019 on patients with acute exacerbation of COPD admitted in the tertiary care FJ Chest hospital, a university teaching hospital in district Quetta, Pakistan.

The study participants were those patients admitted with acute exacerbation COPD, and interviewed by a qualitative questionnaire after obtaining an informed consent approved by the ethical committee. The interview was conducted on those patients who were discharged and ready to go home with an effective way to start a conversation about their medication including the inhaler technique. Seven steps of correct inhaler technique were explored by asking the patient “Can you show me how you use your inhaler in routine?”. Objective inhalation technique assessment was evaluated by looking at the number of steps completed by following the correct sequences of the steps that were recorded. Preferences with different types of inhalers were assessed by asking different questions like whether they had received information about the correct inhaler technique, their preferences, and the technique that had been checked by visiting doctors. Their socio-demographic characteristics, knowledge, and perceptions about their routine inhalers were recorded on the questionnaire. All these observations were recorded at a single cross-sectional visit of study enrolment.

## Results

**Figure 1:**
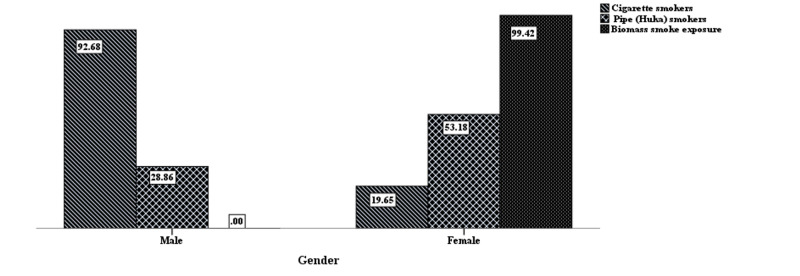
Gender distribution of tobacco smoking and biomass smoke exposure

The study included 765 participants of mean age 58.7 years (SD ±7.8); 32% males and 68% females. Almost all of the females were exposed to biomass fuel smoke exposure (99%) and pipe (Huka) smokers 53%, while most male participants were cigarette smokers (92%) as shown in Figure 1.

**Figure 2:**
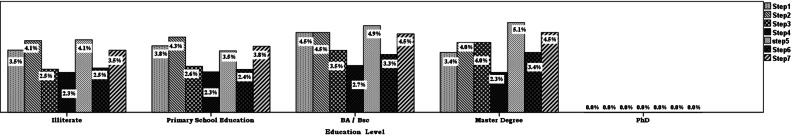
Impact of education level on the correct use of inhaler technique

Overall, 6.3% of patients completed the seven correct steps of both metered-dose inhaler (MDI) and DPI. Few educated participants with a Master's degree and of younger age correctly performed almost seven steps (Figure 2). Both inhalers were being used for a mean duration of 5.3 years (SD ±2.1). MDI was used by 34% without spacer (55%) and DPI was used by 66%. Both MDI and DPI inhaler devices use were associated with multiple errors which included difficulties of pre-inhalation expiration (step-3), maximal inhalation (step-5), and post inhalation breath-holding (step-6) (Table 1).

**Table 1:** Errors associated with MDI & DPI inhaler devices use

Steps	Meter Dose Inhaler (MDI)	Dry Powder Inhaler (DPI)
Step- 1^1^	(32.87%)	(58.37%)
Step -2^1^	(13.78%)	(28.79%)
Step -3^1^	(90.3%)	(88.4%)
Step -4^1^	(0.5%)	(0.3%)
Step -5^1^	(85.9%)	(94.7%)
Step-6 ^1^	(89.1%)	(87.6%)
Step -7^1^	(55.86%)	(34.8%)
1. Jane Scullion, Monica Fletcher. UK Inhaler Group working group. 2018; 1-8. www.respiratoryfutures.org.uk/programmes/uk-inhaler-group. Step1: Prepare the inhaler device Step2: Prepare or load the dose, Step3: Breathe out, fully and gently, but not into the inhaler, Step 4: Place inhaler mouthpiece in the mouth and seal the lips around the mouthpiece, Step 5: Breathe in: pMDI: slow and steady, DPI: quick and deep, Step 6: Remover inhaler from the mouth and hold the breath for up to 10 seconds. Step 7: Wait for a few seconds then repeat as necessary.		

Fifty-four percent of participants were not given the choice of inhaler by the visiting physicians. Interestingly, 47% were using a combination of both inhalers - DPI and MDI, thereby, reducing their symptoms of dyspnea by DPI in 83% of the participants; 73% reported cough relief by MDI use. 38.4% and 15.8% of participants reported that inhaler technique training was provided by pharmacy sales representatives and prescribing doctors, respectively (Table 2).

**Table 2:** Health care personals involved in teaching inhaler technique and their association with inhaler use errors by patients

Health Personals		Inhaler Technique Teaching	Patient’s Inhaler Technique	
			Correct use	with Errors
	Pharmacy Salesman	294 (38.4%)	09 (19%)	285 (40%)
	Pharmacist	201 (26.3%)	03 (7%)	198 (27%)
	Doctors/Consultants	121 (15.8%)	23 (48%)	98 (14%)
	Staff Nurse	93 (12.2%)	07 (14%)	86 (11%)
	Post Graduate Residents	40 (5.2%)	06 (12%)	34 (5%)
	Spirometry Technician	16 (2.1%)	0%	16 (3%)
	Total	765 (100 %)	48 (6.3%)	717 (93.7%)

Fifty-four percent of the participants reported poor adherence to regular use of inhalers as they understand that these inhalers are not effective lately to relieve their symptoms, while 33% were using their inhaler as a maintenance therapy which is quite a low figure. 75% were not aware of any side effects associated with the regular use of inhalers. Lastly, the participant’s education level had an impact on the performance of correct steps, as seen in Figure 2.

## Discussion

This cross-sectional study provides insights on inhalation devices types for delivery of COPD medications, including errors associated with its operation and factors to consider in matching a device to an individual patient. Currently, there are a large variety of inhalation devices available that presents numerous challenges both to patients and prescribing physicians as well [8]. Inhaled therapy represents the mainstay of COPD, therefore, appropriate use of inhaler devices is crucial to mitigate the risk of sub-optimal drug delivery and the subsequent risk of severe flare-ups, hospitalizations, and COPD related outcomes [9-10].

A previous systematic review has found that there has been no change in the types and number of errors reported over the past 40 years [10], and we evaluated errors associated with both DPI and pMDI as shown in Table 1. Inhaler device adherence is relatively poor with approximately 25%-46% of patients remaining adherent to maintenance therapy [11] but in this cross-sectional analysis, the adherence was 33% with poor technique showing the patients belief and trust on their prescribed inhaler relieved their subjective symptoms of dyspnea in 83% and cough in 73% of the participants. Previously, it had been observed that a comprehensive range of reasons for poor inhaler technique were noted, ranging from the device types, the patient preferences, the healthcare provider education on devices use technique, and the technology of inhalers [12]. In this certain group of COPD patients, we observed that most elderly patients could not handle their inhalers and preferred to use nebulizers (14%) which does not involve perception to prepare and efforts requiring to complete the optimal seven steps [13]. Recent data show that 73% of patients consider their inhaler technique to be good or excellent, 86% of patients consider their inhaler easy to use, and compared with our study, 83% participants showed confidence in their inhaler technique quite effectively and had no idea if it required more practice to improve the correct use. Most of the participants had not used their inhaler with all seven optimal steps wrongly handled the devices while performing the crucial steps 3, 5, and 6 for both DPI and MDI inhaler devices (Table 1).

To achieve optimal outcomes for patients with COPD, it is necessary to provide an inhaler that is simple and intuitive to use and is minimally affected by a patient’s technique to ensure maximum efficacy [13]. Fifty-four percent of our patients were not given options as to which type of inhaler they prefer with proper technique demonstration on their clinical visits to respiratory and general physicians. On the other hand, it has been observed that for the last couple of decades, the majority of patients have not been playing their role in using the given device correctly which is multi-factorial [8]. But in this observational analysis, most of the participants with poor technique were elderly as their perception was weak and found it difficult to understand the proper technique; they preferred using nebulizers 14% [13]. A high proportion of patients inaccurately believe that their technique is adequate [14], and as in this study, 83% of participants believed their technique is almost correct. This kind of attitude appears to create a disconnect between inhaler technique theory and practice, as mastery at the time of teaching does not translate into the maintenance of correct inhaler technique over time [15]. We recognize that, for the patient, there is a range of competing factors that impact their willingness, ability, and preference to using their inhalers [16-17], but we believe they can be trained successfully to use their inhalers with effective and repeated instruction [9-18].

Park et al. presented data on changing device switching from a DPI device to a pMDI on the persistence of medication use and effectiveness [19]. In our study, we observed this option was not given to switch from DPI to MDI as the participant's preferences for device selection was not inquired or valued by the visiting physician (55%). Interestingly, it was revealed that DPI (66%) is the most commonly used device as a reliever medication during exacerbation and for maintenance treatment, both DPI and pMDI were used together (47%). Unfortunately, 50% to 81% of patients do not use their inhalational devices accurately, in particular, older patients preparing the dose in the wrong orientation which are the common errors [20-21]; in our study, most of the participants were older patients who could not perform the crucial step 3, 5 and 6 correctly. Few observational studies are more concerned about many health professionals do not use inhalers accurately [22-23] and their role being evaluated by a few countries [24]. Therefore, health care providers are not in a position to assess and coach patients' inhaler technique effectively as noted in Table 2; pharmacy salesman is the main trainer (38%) who educates patients on inhaler technique but with major errors, while 16% reported that their prescribing doctors taught them the technique with less errors while performing their inhaler technique with optimal seven steps. There should be a consensus on who should provide the training to increase patient confidence, and whether patients should be allowed by clinicians to play a more extensive role in device selection. Health literacy is associated with poor use of healthcare services and these observations hold true for patients with COPD as well [25]. Since most of the participants were illiterate (86%), their low literacy rate was associated with using a poor technique with multiple errors (Figure 2).

It is challenging to find the right inhaler device that tailors the patient’s needs, preferences, and ability to use it correctly with minimal errors [26]. This widespread problem coupled with an awareness of device-specific and patient-specific variables affecting inhaler use, may improve clinical outcomes in the management of COPD patients [27-28]. We understand it requires an individualized approach, continued efforts, and repeated instructions with an appropriate device selection. Therefore, an effective training educational programme is required at the national level for proper inhaler use techniques that could minimize the risk of errors. We understand that inhaler handling with optimal steps training could improve adherence and clinical outcomes in COPD patients which could reduce the economic burden on the healthcare system of our developing country.

## Conclusions

This study has shown that poor inhaler technique was highly prevalent among the participants and the errors did not appear to be dependent on inhaler device type (MDI vs. DPI). The participants were not involved in decision making about the choice of inhalation device and education level has an impact on the correct inhaler use. Both MDI and DPI inhaler devices were associated with multiple errors that included difficulties of pre-inhalation expiration, maximal inhalation, and post inhalation breath-holding steps. Therefore inhaler teaching and training is highly recommended with primary focus on the optimal steps errors to address the correct inhaler technique use in the management of COPD patients.
